# Immobilization of Papain in Chitosan Membranes as a Potential Alternative for Skin Wounds

**DOI:** 10.3390/pharmaceutics15122649

**Published:** 2023-11-21

**Authors:** Anne Emmanuelle Câmara da Silva Melo, Felipe Sanderson Ribeiro de Sousa, Alaine M. dos Santos-Silva, Ednaldo Gomes do Nascimento, Matheus F. Fernandes-Pedrosa, Caroline Addison Carvalho Xavier de Medeiros, Arnóbio Antônio da Silva-Junior

**Affiliations:** 1 Laboratory of Pharmaceutical Technology and Biotechnology, Department of Pharmacy, Federal University of Rio Grande do Norte, UFRN, Gal. Gustavo Cordeiro de Farias, Petrópolis, Natal 59072-570, Brazil; annebio@gmail.com (A.E.C.d.S.M.); alaine.maria@hotmail.com (A.M.d.S.-S.); mffpedrosa@gmail.com (M.F.F.-P.); 2 Department of Pharmacology and Biophysics, Federal University of Rio Grande do Norte, UFRN, Lagoa Nova, Natal 59072-970, Brazil

**Keywords:** wound dressing, papain, immobilization, chitosan membrane, drug delivery systems

## Abstract

Papain (an enzyme from the latex of *Carica papaya*) is an interesting natural bioactive macromolecule used as therapeutic alternative for wound healing due to debridement action in devitalized or necrotic tissues. However, its use in high doses can induce potential skin irritation and side effects. In this study, experiments explored the ability of chitosan membrane to immobilize papain, consequently improving enzymatic activity and controlling enzyme release. Papain-loading capacity was tested via experiments of force microscopy (AFM), scanning electron microscopy (SEM-FEG), and X-ray diffraction analyses. Fourier transform infrared spectroscopy and thermal analyses assessed the enzyme interactions with the copolymer. The investigation of the feasibility of membranes included pH on the surface, elasticity, and breaking strength measurements. The surface wettability and swelling capacity of different formulations revealed the best formulation for in vitro papain release experiments. The membranes had a transparent, rough, crystalline characteristic, which was homogeneous with the membrane within the neutrality. The immobilization of papain in the chitosan membrane resulted in a decrease in the vibration band characteristic of pure papain, suggesting a displacement in the vibration bands in the FTIR spectrum. The presence of papain decreased hydrophobicity on the surface of the membrane and disturbed the membrane’s ability to swell. Chitosan membranes containing papain 2.5% (0.04 g) and 5.0% (0.08 g) preserved feasible properties and improved the enzymatic activity compared (0.87 ± 0.12 AU/mg and 1.59 ± 0.10 AU/mg) with a free papain sample (0.0042 ± 0.001 AU/mg). Concentrations of over 10% (0.16 g) led to phase separation into membranes. Chitosan membranes exhibited a slow papain release behavior adjusted via the Higushi model. The experimental achievements suggest a novel and promising method for the enhancement of papain. The results indicate the potential for prolonged bioactivity for use on wounds.

## 1. Introduction

The skin is the largest organ which acts as a natural protection barrier for organisms [1]. Skin damage leads to the appearance of wounds, which serves as an entrance for microorganisms and can result in potential health complications. The healing of skin wounds involves a complex process that consists of a progression of different steps. Wound repair is a crucial field in medicine and the pharmaceutical industry that involves different strategies [2]. In recent years, there have been more than 6.5 million patients with acute and chronic wounds worldwide [3]. Chronic wounds have become a global health problem since the average age of human beings has increased and since the emergence of several complex factors that lead to wounds [4]. Diseases such as diabetes, cardiovascular and cerebrovascular diseases, hypoxia, cancer, immunosuppression, local vascular disease, infections, and repetitive trauma are common causes of chronic wounds [5].

Diabetes is a chronic disease with global impact, mainly type 2 diabetes mellitus. At the rate of its current growth, it is estimated that more than 700 million individuals will be compromised with this disease by 2045 (7.8% of the global population) [6,7]. The traditional treatment for these cases is the use of a passive dressing that protects the damaged area from bacterial infection and provides a suitable environment to accelerate the healing process [8].

Desirable characteristics of dressings include biocompatibility, biodegradability, and non-toxicity to skin. These characteristics are more likely to be provided by natural polymers due to their similarities to the extracellular matrix (ECM) [9]. Materials with these characteristics that can be used include chitosan, collagen, bacterial cellulose, fibroin, and alginate [10]. In addition, bioactive dressings may contain substances with endogenous activity, such as antimicrobials (antimycotics) and chemical or enzymatic deoxidizers [11].

Papain is a proteolytic enzyme found in the latex of *Carica papaya*. It is widely used in the pharmacological field as a debridement agent in the treatment of wounds, lesions, ulcers, and burned skin without causing adverse effects [12]. Papain is already available on the market in the form of ointment and gels for healing wounds, which can be painful and cause friction injuries. The advantage of using this active pharmaceutical ingredient (API) in dressings is that it is easy to apply and remove, providing greater comfort to the patient and prolonged drug action [13]. However, free papain can undergo deactivation when exposed to adverse environmental conditions, favoring its deactivation. To solve this problem, the immobilization of the enzyme on a polymeric support is used [11].

Chitosan is a polysaccharide from chitin, present in the exoskeleton of arthropods and other organisms [14]. Chitosan is rich in hydroxyl and glucosamine groups, which can promote immobilization when associated with enzymes [13,14]. Thus, papain immobilized via electrostatic interactions with chitosan is an interesting and promising strategy to slow enzyme release into the physiological environment of the wound, reaching the most internal tissue layers of the lesion and aiding in the debridement of foreign bodies and cellular waste [15,16]. Enzyme immobilization conditions depend on several factors, such as the type of enzyme, pH, temperature, and ionic strength. The modulation of these factors may induce high enzymatic activity as a support. Surface response methodology is a useful technique to investigate the effects of these independent variables in isolation and in combination for enzymatic immobilization in order to optimize the enzyme immobilization process [17].

Chitosan membranes are especially useful as biomembranes in wound healing applications due to their ease of manufacture and good stability [18]. Chitosan membranes with incorporated papain appear as an alternative delivery device for drugs delivered via topical application. This biomembrane-based dressing creates the possibility of tailoring enzyme activity according to their chemical and physical properties, which can control debridement properties in acute and chronic skin wounds. In this study, experiments explored the ability of the chitosan membrane to immobilize papain and the consequent improvement of enzymatic activity and the control of enzyme release.

## 2. Materials and Methods

### 2.1. Materials

Low-molecular-weight chitosan (code 448859), citric acid (C_6_H_8_O_7_, code 251275), dibasic sodium phosphate (Na_2_HPO_4_, code 94046), ethylenediaminetetraacetic acid disodium salt (EDTA, C_10_H_14_N_2_Na_2_O_8_∙2H_2_O, code E4884), L-cysteine monohydrate (C_3_H_7_NO_2_S∙HCl∙H_2_O, code C7880), casein from bovine milk (code C3400), and trichloroacetic acid (C_2_HCl_3_O_2_, code T6399) were purchased from Vetec™/Sigma-Aldrich, São Paulo, Brazil. Pure papain (code P.10.0801.000.00) was purchased from Dynamica (São Paulo, Brazil).

### 2.2. Characterization Chitosan

The analysis carried out in the study was conducted through the rheological method using the Mark–Houwink–Sakurada equation, quantifying the molecular weight of chitosan molecular mass of 4.61 × 10^5^ g·mol^−1^. The conductivity method evaluated the degree of chitosan deacetylation, which was 88.16% [19].

### 2.3. Preparation of Free Papain Chitosan Membrane

**Figure 1:**
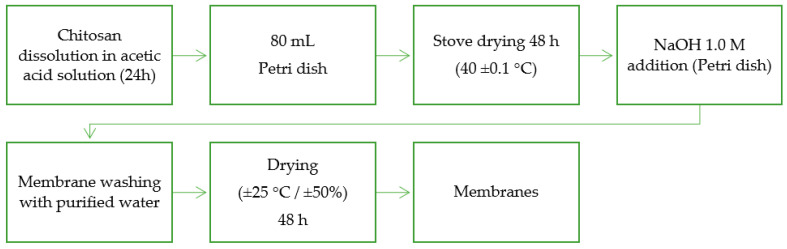
Schematic representation of preparing procedure of chitosan membranes.

For the preparation of the chitosan membrane (Ch membrane), the adapted methodology described in Figure 1 was followed [20]. For an 80 mL (volume) solution: 1.6 g chitosan (2% *w*/*v*) was weighed into 2% *w*/*v* acetic acid. This solution was left under magnetic stirring overnight for homogenization. This solution was poured into Petri dishes (15 cm) and placed in an oven at 40 ± 0.5 °C for 48 h for solvent evaporation. After evaporation, these membranes were neutralized and demolded with 100 mL of 1.0 M NaOH (sodium hydroxide) for 10–15 min. After demolding, the membranes were washed with 250 mL of purified water to remove excess NaOH. To remove the washing water, the membranes were placed at room temperature and humidity controlled (25 ± 0.5 °C for 48 h of 50%).

### 2.4. Papain Immobilization in Chitosan Membranes

Different and rising masses of 0.04 g, 0.08 g, or 0.16 g of papain (equivalent to 2.5%, 5.0%, and 10% of chitosan) were dissolved in a 2% acetic acid solution; the 1.6% copolymer was inserted. The formulations were under magnetic stirring at 375 rpm for 24 h and at room temperature for total dissolution of chitosan and papain. The preparation of chitosan membranes added to free papain followed the same flow as described in Section 2.3.

### 2.5. Analysis of Scanning Electron Microscopy (SEM) and Atomic Force Microscopy (AFM)

Before analysis, the membranes went through the metallization process, which consists of exposing a sample to gold, in a vacuum atmosphere (80 s) with an amperage of 30 mA in metallization equipment, model SCD 005 Sputter Coater, brand BAL-TEC^®^ (Los Angeles, CA, USA). The equipment used to evaluate the thickness of the membranes was CARL ZEISS^®^, model Auriga (Oberkochen, Germany), using an acceleration voltage of 5 kV. The software used for the evaluation was SmartSEM^®^ Version 05.06. Samples were quantified in sextuplicate (n = 6).

The AFM analysis measured the surface and aspect of the membranes. The equipment used for sample measurements was AFM, SPM-9700, Shimadzu^®^ (Tokyo, Japan). The temperature was 25 °C, with the cantilever without contact, and the frequency was 70 Hz, with a constant force of 1 to 5 N/m.

### 2.6. pH Analysis of Membranes

Each membrane sample was immersed in 10 mL of PBS (pH = 7.4) for 1 min. The excess was removed with filter paper and subjected to pH measurement. The pH meter model was Skin Equipment, brand COURAGE+ KHAZAKA^®^ (Cologne, Germany). The samples had a size of 16 mm^2^ and the repetitions occurred in triplicate (n = 3) [21].

### 2.7. Mechanical Properties

Mechanical properties were analyzed by elasticity and breaking strength. The samples were prepared in rectangles with the size of 100 mm × 10 mm. The samples underwent axial extension. The membrane was coupled to an extender device, automatic in the universal mechanical testing machine EMIC DL10000 (Paraná, Brazil) according to ASTM D638. All analyses were performed in sextuplicate (n = 6) [22].

### 2.8. Fourier Transform Infrared (FTIR) Spectroscopy

The samples were submitted to Fourier transform infrared spectroscopy analysis with attenuated total reflection accessory (FTIR-ATR). The model used was the IR-Prestige-21, Shimadzu^®^ equipment (Tokyo, Japan). The MID (middle Infrared) spectral region ranged from 700 to 4000 cm^−1^, with a resolution of 4.0 cm^−1^.

### 2.9. X-ray Diffraction (XRD) Analyses

The apparatus used for X-ray diffraction analysis was XRD 7000 Shimadzu^®^ (Tokyo, Japan). The parameters used were an angle of 0.02° and a sweep speed of 2 degrees/min. The samples were run through a copper tube with a current of 30 mA and a voltage of 40 kV. XRD graphs were plotted with the 2θ variation from 5° to 40°.

### 2.10. Thermal Analyses

The model used for differential scanning calorimetry (DSC) thermal analysis was model DSC-210, Shimadzu^®^ (Tokyo, Japan). For sample preparation, 4 mg of membranes were weighed in platinum containers, duly sealed. The samples were subjected to a temperature of 10 °C/min ranging from 30 to 400 °C, over N_2_ at a rate of 50 mL/min. Before the analysis, the equipment was calibrated according to the standard (p.f. 156.6 °C and ΔH = 28.45 J/g).

For thermogravimetric analysis (TGA), the equipment used was model SDTQ600, TA INSTRUMENTS^®^ (New Castle, DE, USA). In total, 5 mg of the sample was weighed in the alumina crucible, using it under the same conditions as in the DSC analyses, with a temperature variation from 30 to 900 °C. Derivations of thermogravimetric analysis (DTG) aimed to visualize Tonset thermal events.

### 2.11. Swelling Assay and Water Contact Angle Measurements

Each membrane segment reported in Section 2.7 was immersed in PBS (pH = 7.4). Samples were weighed at the following specific intervals: 0.5, 1, 2, 3, 6, 12 and 24 h. After removing the sample from the PBS, it was transferred using filter paper for 1 min, and its weight (mass) was quantified. The percentage of water absorption (swelling) was expressed using the following equation: swelling (%) = [(Wt − Wo)/Wo] × 100, where Wt is the mass of the sample at the specific time interval t, and Wo is the mass of the sample before the experiment.

The samples were placed on the flat, mobile base in a triplicate membrane; then, a drop of 10 μL of distilled water was deposited. The images were captured with a micro camera and calculation of the contact angle was performed. The angle formed by the liquid was assessed in time intervals (t) of 10 in 10 s, with the beginning t = 0 s and end t = 60 s. The software used to isolate the images was Pinnacle Studio Quickstart^®^ version 8. Each time, five measurements were performed from which mean and standard deviation were determined. The software used to calculate the contact angle was surftens^®^, Universal basic version.

### 2.12. Enzymatic Activity Assay

The proteolytic activity of papain was determined as previously described based on the universal protocol of protease activity developed by Sigma-Aldrich [23]. The mixture of 0.5 mL of an activator solution was prepared in phosphate buffer (pH = 8) containing 0.038 mol·L^−1^. EDTA and 0.034 mol·L^−1^. L-cysteine hydrochloride monohydrate was added to 0.5 mL of 2% (*w*/*v*) papain. After incubation for 10 min at 37 °C, 2 mL of 1% (*w*/*v*) casein prepared in phosphate buffer (pH = 8) was added to the mixture. Substrate hydrolysis occurred during 10 min at 37 °C. Casein proteolysis was stopped by the addition of 2 mL of 10% (*w*/*v*) trichloroacetic acid.

The activity of immobilized papain in the chitosan membrane was measured in the same way as for the free enzyme. The samples were centrifuged at 10,000 rpm for 10 min and the supernatant was collected; it was measured at 660 nm using a model UV-2450 spectrophotometer (Shimadzu, Tokyo, Japan). Blank tests were performed with the addition of trichloroacetic acid prior to the addition of the papain solution. The amount of tyrosine released after hydrolysis was used to calculate the proteolytic capacity of papain in different samples. Each enzyme unit was defined as the amount of enzyme that hydrolyzes casein to produce an absorbance equivalent to 1 μmol of tyrosine·min^−1^. The correlation between absorbance and amount of tyrosine (μmol·mL^−1^ of deionized water) was used to plot the standard curve.

### 2.13. In Vitro Papain Kinetic Release Studies

For the analysis of the papain release system incorporated into chitosan membranes, Franz-type diffusion cells were used. The in vitro release study was conducted with vertical Franz diffusion cells with a capacity of 15 mL and 1.8 cm^2^ of diffusion area. The receiver compartment was filled with 0.1 mol/L of phosphate-buffered solution (pH 7.4 at 37 ± 0.5 °C). Collections took place at specific intervals (0.5, 1, 2, 3, 4, 6, 8, 12 h); 1 mL of dissolution medium was subjected to the determination of papain through quantification by the Lowry method [24]. Fresh buffer volume was immediately replaced after each measurement. Kinetic drug release data can be subjected to different mathematical models to compare the formulations [25,26,27,28]. In the present study, a flat device, a specific membrane, was tested by using Franz-type diffusion cells and the well-established Higuchi’s model was applied. The drug release is diffusion-based on Fick’s Law, expressed as a function of square root of time through equation *Mt*/M∞ = *kt* 0.5. *Mt*/*M∞* is the fraction of released drug, *t* represents the interval of measurement, *k* is the constant of release rate, and *n* is the diffusional exponent suggestive of a transport mechanism.

### 2.14. Statistical Analysis

The collected experiment data were expressed as means of experimental values and their respective standard deviations. Statistical analysis between the two groups was performed using the Student’s *t*-test, and multiple groups were evaluated by one-way ANOVA followed by the post hoc Tukey test using SPSS, Version 26. A value of *p* ≤ 0.05 was considered statistically significant. Experimental assays were performed at least in triplicate.

## 3. Results and Discussion

### 3.1. Preparation of Membranes and Morphology

**Figure 2:**
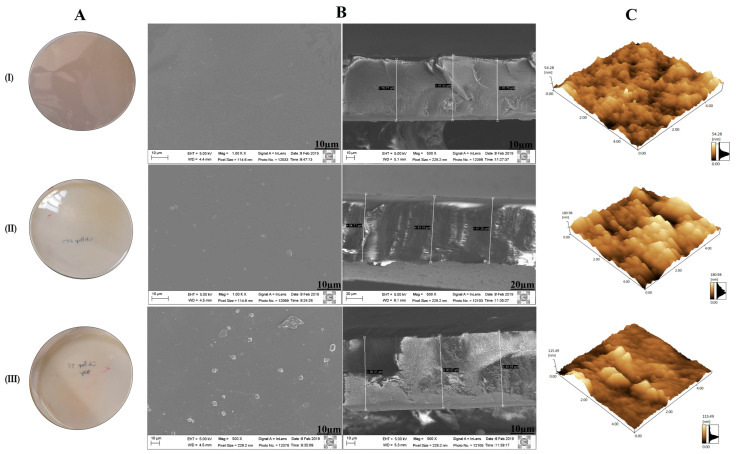
(**A**) Optical image, SEM images. (**B**) Top view SEM images and sectioned samples. (**C**) 3D AFM images of different samples. (I—free papain chitosan membrane, II—chitosan membrane containing 2.5% of papain, and III—chitosan membrane containing 2.5% of papain).

Four different chitosan membrane formulations (membrane Ch, ChPap2.5%, ChPap5% e ChPap10%) were prepared. One free papain (membrane Ch) and three papain-loaded chitosan membranes containing 2.5% *w*/*w* (membrane ChPap2.5%), 5.0% *w*/*w* (membrane ChPap5%), and 10% *w*/*w* of enzyme (membrane ChPap10%), respectively, were used. However, formulation membrane ChPap10% proved to be brittle and with phase separation, and it was excluded in further steps. Figure 2 shows optical, SEM, and AFM images for the three formulations, membrane Ch (control), membrane ChPap2.5%, and membrane ChPap2.5%.

The (I) free papain chitosan membrane (membrane Ch), used as a control, proved to be transparent and flexible membrane. Figure 2A shows the images of different samples of membranes. The SEM images of the top view and sectioned membrane (Figure 2B) showed a uniform membrane, without particles on the surface and no phase separation. In addition, the 3D AFM images showed a slight surface roughness (Figure 2C). The rising of papain loading of (II) 2.5% to (III) 5.0% perturbed membrane formation. Therefore, it was possible to observe particles on the surface, but no differences in the sectioned SEM images were seen (Figure 2B). In addition, papain loading seemed to decrease the roughness of the surface (Figure 2C).

### 3.2. Surface pH Measurements and Mechanical Properties

All the formulations had their surfaces subjected to pH measurements (Table 1). The free papain chitosan membrane (membrane Ch) showed a slight neutral pH of about 7.1, while papain loading increased to values of about 7.5 (membrane ChPap2.5% and membrane ChPap5%).

### 3.3. Papain Interactions with Chitosan by X-ray Diffraction, Thermal Analyses and FTIR

**Figure 3:**
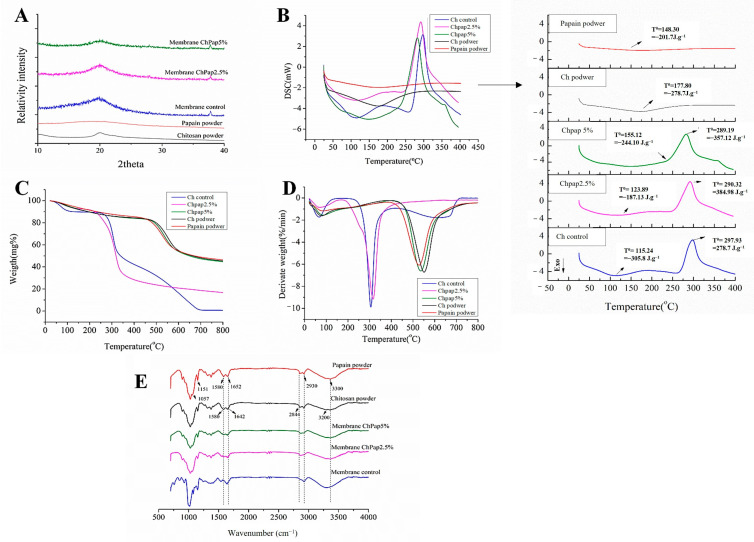
(**A**) XRD analyses, (**B**) DSC curves, (**C**) TGA curves, (**D**) DTG curves, and (**E**) FTIR spectra for different samples. Note: black line: chitosan, red line: papain, blue line: free papain chitosan membrane (membrane Ch), pink line: chitosan membrane containing papain 2.5% (membrane ChPap2.5%), and green line: chitosan membrane containing papain 5% (membrane ChPap5%).

Figure 3A shows X-ray diffractograms of different samples. Papain (pap) exhibited a characteristic amorphous character, while chitosan (Ch) showed characteristic peaks of semi-crystalline copolymer, recorded at about of 2θ = 10° and 2θ = 20°, respectively. Figure 3B shows DSC curves for different samples. Any phase transition identified for papain corroborated its amorphous character. The amorphous character of papain (red line) was identified by the absence of any phase transition. Figure 3B shows the DSC curves for different samples. Chitosan (black line) exhibited a characteristic event of dehydration before 100 °C, confirming the TG (Figure 3C) and DTG (Figure 3D) curves. All formulations containing chitosan presented two thermal degradation events. In the powder Ch, there was the first event at 66.75 °C and a loss of 4.5%, and the second event at 304.60 °C with a loss of 68.52%. In membrane Ch 52 °C, the first event had a loss of 3% and the second event occurred in 278 °C with a loss of 68% of mass. In ChPap2.5% at 52 °C, there was a loss of 3.2%, and at 287.14 °C, there was a loss of 67.12%. In ChPap5% at 56 °C, there was a loss of 2.4%, and at 280 °C, there was a loss of 68.63%. Papain powder presented only one event at 314 °C with a loss of 54.04% of mass. Figure 3E shows FTIR spectra for different samples.

### 3.4. Water Contact Angle, Swelling Assay, and In Vitro Papain Release

**Figure 4:**
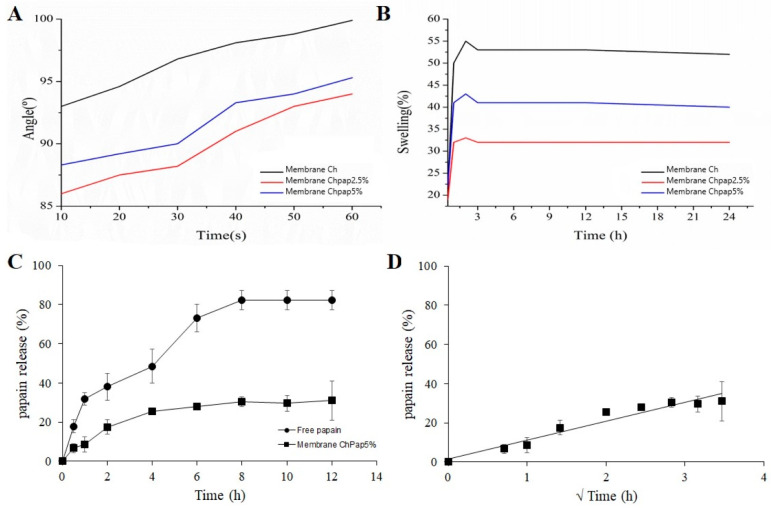
(**A**) Water contact angle as a function of time. (**B**) Swelling rate test. (**C**) Papain in vitro release experiments. (**D**) High-fitted data of papain release from chitosan membrane (membrane ChPap5%).

Figure 4A shows drop shape analysis used to determine contact angle variation as a function of time. The effect of composition and changes in the copolymer interactions for membrane swelling is assessed in Figure 4B. Formulations containing 5% present greater swelling, which have the in vitro papain release assessed. Figure 4C shows the in vitro release behavior for free papain and immobilized papain on the chitosan membrane (membrane ChPap5%), while Figure 4D shows the Higushi plot.

### 3.5. Enzymatic Activity Performance

Finally, free papain and different formulations have enzyme activity as assessed to predict potential in vivo efficacy before and after immobilization. The free papain exhibited enzymatic activity of 0.0042 ± 0.001 AU/mg, while chitosan membranes containing 2.5% and 5% presented, respectively, 0.87 ± 0.12 AU/mg and 1.59 ± 0.10 AU/mg. This achievement suggested that chitosan membranes containing 2.5% and 5% improve papain activity, which was about 200 and 380 times higher than that of pure papain.

## 4. Discussion

The differences identified in the surface of papain-loaded membranes can induce distinct dermal dressing effects, considering roughness. They can allow the exchange of gas deposition between the membrane and the external environment, favoring wound oxygenation and bioadhesion. This certainly changes the cell proliferation and differentiation, such as fibroblasts, osteoblasts, and production of extracellular matrix components, facilitating healing. Authors claim that chitosan membranes incorporated with different drug concentrations may interfere with the appearance of color becoming darker with increasing drug concentration. But in the SEM images (Figure 2), there were no differences in the external appearance: they were all very smooth and uniform, suggesting that papain is well distributed in the chitosan matrix, corroborating the findings found in our studies [29,30,31]. A similar effect was observed when glycerol and allantoin were entrapped in chitosan membranes [32]. The tested compositions with papain (2.5% and 5.0%) presented as suitable devices for use in medicine as biodegradable membranes for healing action.

Regarding topical application, pH and mechanical properties are also important feasible properties. The identified pH range in Table 1 is considered skin compatible, mainly for healing action. A membrane with a pH that is too acidic or basic affects the area of application and causes damage to the oral mucous membrane, leading to patient discomfort. This study verified that the drug involved in the membrane can affect the final pH of the product, which may impair the healing process of the mucosa [33].

Membranes with variations in pH (strong or alkaline) induce irritation of the mucosa causing discomfort to patients [34]. Considering mechanical properties, papain loading at 2.5% (membrane ChPap2.5%) and 5.0% (membrane ChPap5%) did not affect both tensile strength (TS) and elongation break (%) compared to free papain (membrane Ch). The increment of papain did not statistically decrease the ability of membrane support stress before breaking. Despite fine differences in SEM and AFM achievements, no statistical differences were identified among mechanical properties of three samples. It is expected that considerable drug loading into chitosan membranes could interrupt the intermolecular interactions between its chains. Thus, mechanical strength of the chitosan material mainly depends on the hydrogen bonds between the chitosan structure. Therefore, incorporation of papain in the studied range did not affect or sufficiently disturb these intra- and intermolecular hydrogen bonds to decrease the tensile strength, as reported in previous studies [35,36].

The structural properties and manner in which the different concentration of papain interacts with chitosan in membranes were also assessed by using DRX, DSC, TG and FTIR analyses. The amorphous character of the chitosan membrane (Figure 3A) seems amplified into papain-loaded membranes, suggesting that enzyme loading perturbs the structure of chitosan into membranes. This papain–chitosan interaction is important for controlling the enzyme release behavior. This phenomenon can be better explored assessing possible enthalpy variation in important phase transitions, which suggest changes in the structure of the free and immobilized enzyme in the supports. The calorimetric curves of the samples, with the variation of temperature and enthalpy (ΔH), were recorded (Figure 3B). We can observe that there was an increase in the endothermic peak of the membranes incorporated with papain compared to the control. This possibly occurred due to the cleavage of the intermolecular hydrogen interactions (polar) and van der Waals interactions (non-polar) between papain and the chitosan matrix. For free papain, a slight peak observed before 148 °C was associated with the breakdown of weak interactions in the 3D structure of the protein at this temperature range. The first endothermic event in the papain curve was related to the unfolding of the enzyme structure (denaturation). Similar results were observed in the DSC curves of papain, which showed an increase in thermal stability confirmed by the measurement of the apparent thermal denaturation temperature of papain into the membrane (from 141.8 °C to 151 °C, respectively) [37,38].

The thermal stability of papain is reinforced in TG and DTG curves (Figure 3C,D). This is an interesting achievement, especially considering the enzymatic stability for the healing effect. Endothermic events often occur in organic compounds due to any phase transition such as relaxation, and it is attributed to the melting temperature [39]. Membranes presented an endothermic peak at 100 °C, while chitosan systems with papain increased the thermal stability of the enzyme. This achievement was confirmed by measuring the temperature of thermal denaturation that occurred at superior temperature ranges. These results corroborate those found in anterior studies with alginate and cellulose membranes in which chemical interactions of papain with copolymer can occur by hydrogen bonding and polyelectrolyte complexation [16,40].

The FTIR of isolated chitosan (black line) showed N-H vibrational bands and the O-H functional group in the region between 3570 and 3000 cm^−1^ [41]. An overlap of symmetric and asymmetric C-H bands was observed in the 2955–2845 cm^−1^ range. In the region of 1658–1515 cm^−1^, the typical overlapping vibration of the N-H and C=O bonds was observed. In the region of 1640 cm^−1^, the vibration of the C=O bond belonging to the CH_2_ of the biopolymer occurred; the vibrations of elongation of the C-O-C bond corresponding to the structure of the saccharide at 1154 cm^−1^ and 896 cm^−1^ were also found in this spectrum [32,40,42,43]. Isolated papain (red line) showed a predominant band at 3450–3225 cm^−1^, with a peak at 3300 cm^−1^ due to the N-H stretch of an N-substituted secondary amide [44,45]. The band at 2930 cm^−1^ is typically associated with asymmetric CH_2_ elongation. The two absorption peaks in the bands at 1580 and 1640 cm^−1^ are typical for the amide I and amide II regions. The authors claim that the appearance of peaks in the range of 1500–1700 cm^−1^ is in the spectrum for papain and primary and secondary amines [30,44]. The peaks at 1160 and 1057 cm^−1^ were attributed to the C-S sulfide and disulfide stretch, and strong peaks between 1150 and 1050 cm^−1^ occurred due to the C-S stretch of sulfides and disulfides. Immobilization of papain on chitosan membrane resulted in a decrease in the characteristic vibration band of pure papain, suggesting a shift in vibration bands in the FTIR spectrum. These achievements corroborate papain–chitosan interactions by hydrogen bonding and electrostatic interaction.

Aside from investigating the effect of papain on the intermolecular interactions of chitosan in the membrane, it is important to assess how it affects the surface and hydrophilic properties of the intended biomembrane. Chitosan is insoluble in purified water and soluble in acid pH. Thus, preparation of chitosan membranes is an interesting strategy for tailoring solubility, surface and mechanical properties of this class of copolymers which are dependent on their composition. Water wettability of different formulations of chitosan membranes was studied by water contact angle measurement (Figure 4A). It is interesting to highlight that designated hydrophilic surfaces occur for values lower than 90° to the formed water contact angle [46]. Thus, papain decreased the contact angle for chitosan membranes, suggesting an improvement on the wettability, possibly due to the changes in the roughness of membranes and the presence of a hydrophilic compound on their surface [47].

Following the swelling tests (Figure 4B), free papain chitosan membranes (membrane Ch) showed about 50% of swelling capacity after 15 min, which decreased with papain loading. Although papain increased the hydrophilicity on the surface, its presence on the membrane did not contribute to the increase in the swelling. This achievement corroborates the FTIR analyses, which demonstrated that papain changed the intra- and intermolecular interactions in the chitosan chains, changing its solubility and swelling behavior. Chitosan is a natural polysaccharide with known swelling capacity in aqueous media due to the presence of amino groups characterized by electronegative N-H groups. The desired environment for successful wound healing involves keeping the wound bed moist [10,48]. In addition, wettability affects papain release rate [49]. In the present study (Figure 4C), the chitosan membrane shows slow papain release. Previous studies showed faster papain release from alginate membranes with considerably decreased enzymatic activity [38]. This achievement shows the important hole of this cationic hydrogel for better bioadhesion with anionic cell surfaces on affected tissues.

Mathematical modeling of papain release data from chitosan membrane showed good correlation with the model of Higushi (Figure 4D, *Mt*/*M∞* = *kt* ^0.5^, r = 0.97) [50]. The Higuchi model is one of the mathematical models originally designed to explain drug release from creams or ointments from flatted devices, such as, for example, the Franz cell type diffusion devices. It can be used to describe drug release as a form of diffusion based on Fick’s Law, relating drug release as a function of the square root of time. Thus, it has been well applied to explore drugs or biomolecule release from polymeric films and membranes [51,52]. In addition, the involved Fickian diffusion mechanism explains drug release controlled by swelling of polymeric membranes, which, with partial contact with an aqueous medium, is modulated by concentration gradient [53,54]. It is interesting to report that the healing and remodeling process is slow, and that the slow release properties considering papain determine therapeutic success.

The capacity of chitosan for improving the enzyme activity of papain was also assessed and demonstrated in this study. Previous studies demonstrate enzyme immobilization in chitosan supports, preserving its activity [55]. Protein interactions with chitosan chains changed the structure of the enzyme, increasing its capability to hydrolyze casein into smaller peptides [56]. It is desired that the enzyme must maintain or improve its proteolytic function after immobilization. The evaluation of the proteolytic capacity of immobilized papain was necessary, since the immobilization of enzymes via covalent bonding can decrease the catalytic performance of the protein [57]. In this study, papain immobilization in the chitosan membranes occurred by hydrogen bound and electrostatic interaction, creating a “virtual crosslinking”. This fact changes wettability on the membrane surface and the swelling capacity, preserving the ability to slow the release of the enzyme. Thus, the papain-loaded chitosan membranes presented the desired and promising characteristics to improve the therapeutic efficacy of papain in wound healing.

## 5. Conclusions

Experimental data demonstrated the ability of chitosan membranes for loading papain with considerable improvement on enzymatic activity and the ability to supply a slow-release profile. Enzymatic immobilization proved to be viable at concentrations of 2.5 and 5% of the enzyme. Papain interactions with chitosan chains seem to have occurred by hydrogen bounds, which changed the wettability and swelling behaviors of the membrane. The tested devices showed suitable mechanical properties for application in wound-affected skin. These aspects can be well-modified according to their composition. Therefore, the broad and meticulously experimental approach assessed their physicochemical properties and suggested an innovative and promising device for the slow papain release as a bioactive dressing for the treatment of wound healing. It is known that biomaterials such as chitosan are used to improve the healing process and are also an immobilization support. Papain is a proteolytic enzyme used to enhance wound healing. The use of biological dressings with a biomaterial and a debriding agent may represent an advantageous and easily accessible option for treatment of diabetic wounds.

## Figures and Tables

**Table 1:** Measurements of pH and mechanical properties for different chitosan membrane formulations.

Membrane Formulations	Surface pH	Tensile Strength (N)	Elongation at Break (%)
Membrane Ch	7.1 ± 0.22 ^(a,b)^	33.8 ± 11.15 ^(a,b)^	2.5 ± 3.5 ^(a,b)^
Membrane ChPap2.5%	7.5 ± 0.32 ^(a,b)^	33.2 ± 10.07 ^(a,b)^	2.3 ± 2.8 ^(a,b)^
Membrane ChPap5%	7.4 ± 0.17 ^(a,b)^	20.2 ± 14.34 ^(a,b)^	1.1 ± 1.38 ^(a,b)^

Data are presented as mean ± standard deviation. The ANOVA (^a^) test with Tukey’s post-test (^b^) was used. No statistical differences (*p* < 0.05).

## Data Availability

Data are contained within the article.
